# Exploitation of marine bacteria for production of gold nanoparticles

**DOI:** 10.1186/1475-2859-11-86

**Published:** 2012-06-20

**Authors:** Nishat Sharma, Anil K Pinnaka, Manoj Raje, Ashish FNU, Mani Shankar Bhattacharyya, Anirban Roy Choudhury

**Affiliations:** 1 Council of Scientific and Industrial Research (CSIR), CSIR-Institute of Microbial Technology (IMTECH), Sector – 39A, Chandigarh, 160 036, India

**Keywords:** Marine bacteria, *Marinobacter pelagius*, Gold nanoparticles, Biological synthesis, Polyphasic taxonomy

## Abstract

**Background:**

Gold nanoparticles (AuNPs) have found wide range of applications in electronics, biomedical engineering, and chemistry owing to their exceptional opto-electrical properties. Biological synthesis of gold nanoparticles by using plant extracts and microbes have received profound interest in recent times owing to their potential to produce nanoparticles with varied shape, size and morphology. Marine microorganisms are unique to tolerate high salt concentration and can evade toxicity of different metal ions. However, these marine microbes are not sufficiently explored for their capability of metal nanoparticle synthesis. Although, marine water is one of the richest sources of gold in the nature, however, there is no significant publication regarding utilization of marine micro-organisms to produce gold nanoparticles. Therefore, there might be a possibility of exploring marine bacteria as nanofactories for AuNP biosynthesis.

**Results:**

In the present study, marine bacteria are exploited towards their capability of gold nanoparticles (AuNPs) production. Stable, monodisperse AuNP formation with around 10 nm dimension occur upon exposure of HAuCl_4_ solution to whole cells of a novel strain of *Marinobacter pelagius*, as characterized by polyphasic taxonomy. Nanoparticles synthesized are characterized by Transmission electron microscopy, Dynamic light scattering and UV-visible spectroscopy.

**Conclusion:**

The potential of marine organisms in biosynthesis of AuNPs are still relatively unexplored. Although, there are few reports of gold nanoparticles production using marine sponges and sea weeds however, there is no report on the production of gold nanoparticles using marine bacteria. The present work highlighted the possibility of using the marine bacterial strain of *Marinobacter pelagius* to achieve a fast rate of nanoparticles synthesis which may be of high interest for future process development of AuNPs. This is the first report of AuNP synthesis by marine bacteria.

## Background

Synthesis of metal nanoparticles has become a focus of current interest due to their unique properties which are markedly different from their bulk counterparts. Gold nanoparticles have found wide applications in several areas like optoelectronics, photonics, catalysis, imaging technology, drug delivery, space science etc. due to their stability, resistance towards oxidation and biocompatibility [1-5]. A variety of processes including physical, chemical and biological methods are available for synthesizing metal nanoparticles [6-9]. Gold nanoparticles are conventionally synthesized by reducing a gold salt with sodium borohydride or sodium citrate. It is possible to obtain reasonably good monodispersity and obtain nanoparticles with desired capping ligands by those methods. However, the production of gold nanoparticles via chemical routes involves use of toxic organic solvents and the process is not environment friendly [10]. Moreover, in most of the cases the yield is low and requires stringent downstream processing to obtain monodisperse solution of nanoparticles. Therefore, a strong need has been felt to develop environmentally benign metal nanoparticle production technology. Biological production of metal nanoparticles has been tried as an obvious alternative of the chemical and physical processes, with promises of greener process [11,12]. Understanding of the natural processes will obviously help in the discovery of completely new and unexplored methodology of metal nanoparticle synthesis [13-18].

Despite of our knowledge of metal-microbe interaction, specially pertaining to the bioremediation and bioleaching of metals, only recently, the potential of biological agents like bacteria, fungus and plants have been realized for metal nanoparticle synthesis. In recent times, researchers are concentrating on use of microbes as “nanofactories” for production of metal nanoparticles [19]. Though, in recent time many organisms have been described to produce nanoparticles, scientific understanding on the mechanism and the machinery related to its production is still in its infancy [20-22]. Therefore, the available methodology of green synthesis of nanoparticles of novel metals like gold, silver, nickel etc., is unable to meet the present day scientific and industrial demand. On the other hand, prokaryotes have received maximum attention in the area of metal nanoparticles synthesis. This is probably due to the fact that bacterial cells have the ability to resist environmental stresses and have the capability of growing in presence of high metal concentrations. Gold is one of the most studied metals in terms of biological synthesis of nanoparticles [11,23,24]. In most of the published reports gold nanaoparticles were synthesized using bacteria isolated from metal contaminated soils [25]. Astonishingly, marine-microorganisms have not been exploited for their capabilities of AuNP synthesis though, marine water is known to be the richest source of gold in nature. Therefore, it is important to explore marine microflora for AuNP production.

In a radical shift from the predominantly soil microbe based methods for gold nanoparticles production, we have investigated capability of marine bacteria to synthesize gold nanoparticles. We have screened several marine bacteria isolated from different sea cost in India and obtained one marine bacterium having capability of gold nanoparticles synthesis. The selected marine bacterium has been characterized using polyphasic taxonomy. The gold nanoparticles synthesized using whole cells of the bacterium were further characterized using UV-visible spectroscopy, dynamic light scattering (DLS) and Transmission Electron Microscopy (TEM). In this communication, we present the first report of gold nanoparticles synthesis by marine bacteria. This observation of synthesizing gold nanoparticles by marine bacteria may be considered as the first step towards harvesting gold from sea water, nature’s richest source of gold.

## Results and discussions

**Figure 1:**
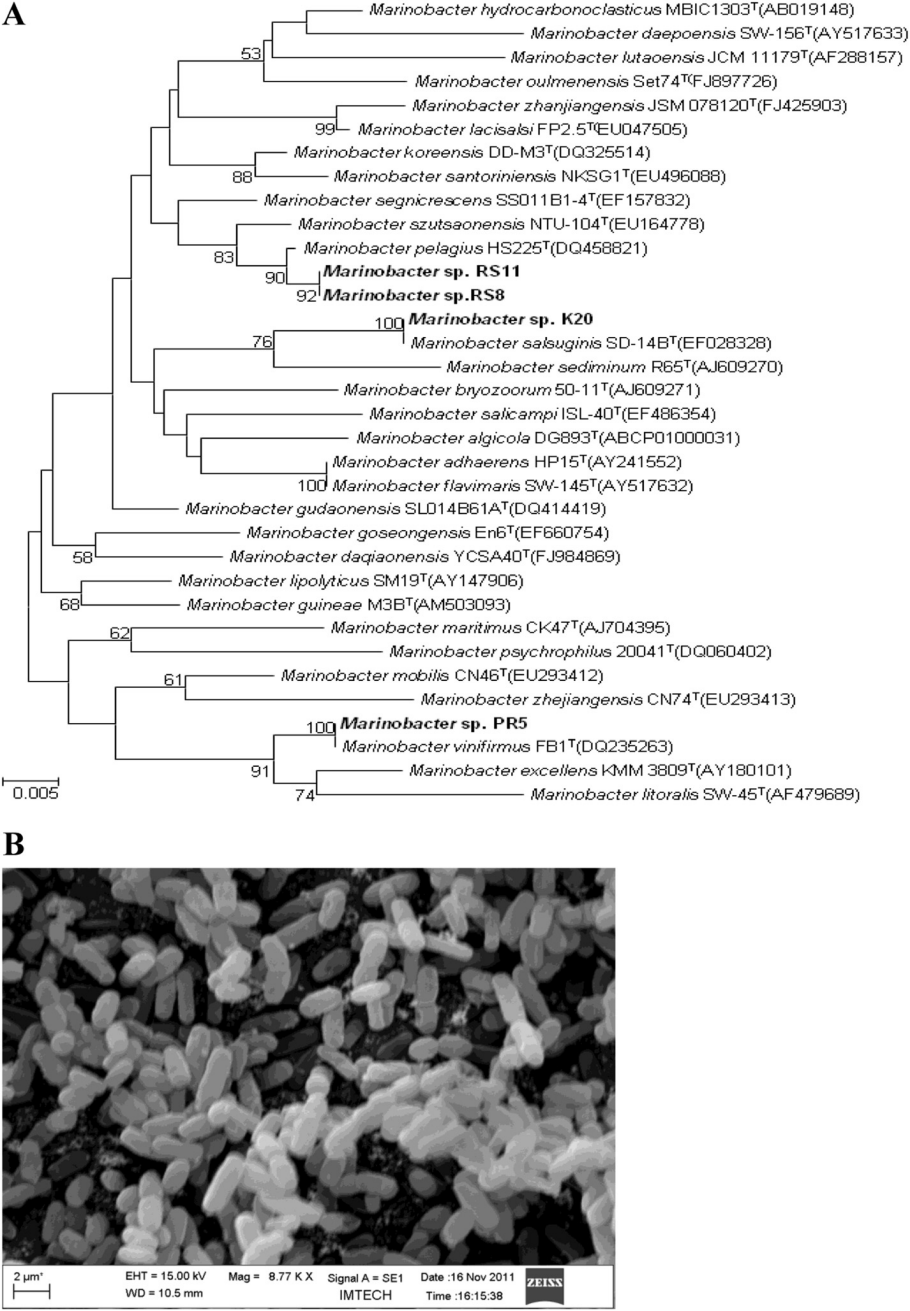
**A: Phylogenetic tree based on 16 S rRNA gene sequences showing the relationship of*Marinobacter*sp.** RS11 with member of the genus *Marinobacter*. Numbers at nodes are bootstrap values ≥50%. Bar, 0.005 substitutions per nucleotide position. **B**: Scanning Electron micrograph of *M pelagius.*

**Figure 2:**
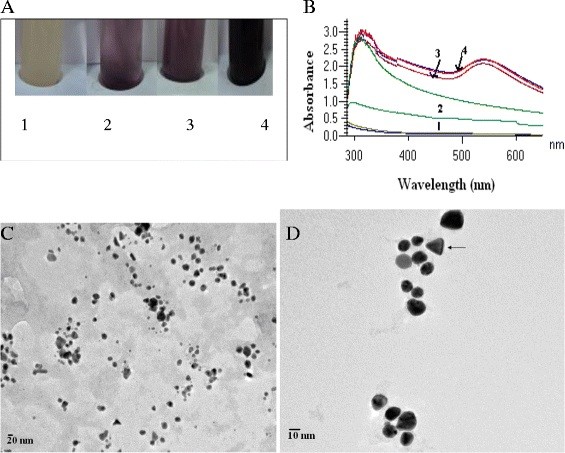
**A: Pictures of test tubes containing the bacteria*M. pelagius*cells before (test tube 1) and during incubation in an aqueous of HAuCl**_**4**_**solution at pH 7.8. B**: UV–Vis absorption spectra of gold nanoparticles after the incubation of *M. pelagius* in 1x10^-3^ M aqueous HAuCl_4_ solution at 7.8 pH. The numbers (1,2,3 and 4) indicates the absorption spectra taken at different time intervals 24,48,72 and 96 respectively. **C, D**: TEM images of gold nanoparticles produces by the reaction of 1x10^-3^ M aqueous HAuCl4 solution with bacteria *M.pelagius* biomass at 7.8 pH. Particles are mostly spherical with nano triangles also present (arrow). Bar = 20 nm (**A**) and 10 nm (**B**).

The strain, RS 11 which is positive for gold nanoparticle synthesis was further characterized by polyphasic taxonomic approach which includes morphological, physiological, biochemical and phylogenetic analysis. The phylogenetic relationship of strain RS11 was analyzed based on the 16 S rRNA gene sequence similarity of RS11 with the related species using BLAST sequence similarity search (EzTaxon). The results indicated that strain RS11 was close to *Marinobacter pelagius* JCM 14804^T^ with the pair wise sequence similarity of 99.6%. Phylogenetic analysis based on maximum likelihood and neighbour joining trees indicated that strain RS11 clustered with the species of the genus *Marinobacter***(**Figure 1A). Strain RS 11 was found to be gram negative, non-endospore forming rods **(**Figure 1 B**)** and the colonies were circular, 3–4 mm in diameter, creamish and convex with entire margin after two days of growth on marine agar at 30 °C. The strain grew between 20^o^ to 45^o^ C temperature, optimum being 30 °C to 37 °C and from pH 6 to 10, with the optimum growth at pH 7.0-8.0. Growth occurred at salinities from 0.5 to 8% (NaCl, w/v), with an optimum between 2-4% NaCl (w/v). In an effort to isolate and screen marine bioresource for AuNP synthesis eleven unique morphotypes were selected from the serial diluted plates and were identified by 16 S rRNA gene sequence analysis (Table 1**)**. These eleven strains were screened for their capability towards production of gold nanoparticles. Among those selected strains, RS 11 was found to synthesize gold nanoparticles visualized by the colour change of the solution from yellow to pink **(**Figure 2**A)**. This formation of AuNPs was also confirmed by the presence of characteristic surface plasmon resonance of gold nanoparticles at 540 nm **(**Figure 2**B)** measured by UV- Visible spectrophotometer.

**Table 1:** List of the marine isolates screened for the gold nanoparticle synthesis

**S.No**	**Strain**	**Culture**	**Source of isolation**
1	RS8	*Marinobacter pelagius*	Water sample from solar saltern, Kakinada
2	*RS11	*Marinobacter pelagius*	Water sample from solar saltern, Kakinada
3	RS15	*Halomonas ventosae*	Water sample from solar saltern, Kakinada
4	LD4	*Pseudoalteromonas mariniglutinosa*	Surface sea water from Lakshadweep
5	LD14	*Pseudoalteromonas mariniglutinosa*	Surface sea water from Lakshadweep
6	LD16	*Alteromonas* sp.	Surface sea water from Lakshadweep
7	LD17	*Alteromonas macleodii*	Surface sea water from Lakshadweep
8	PR5	*Marinobacter vinifermus*	Sea water sample from srilanka coast
9	K4	*Halomonas ventosae*	Algal mat sample from mangrove forest of Namkhana, West Bengal
10	K20	*Marinobacter salsuginis*	Sediment sample from Mangrove forest, Namkhana, West Bengal
11	M6	*Photobacterium ganghwense*	Sediment sample from southeast coast, Palk Bay, India

**Figure 3:**
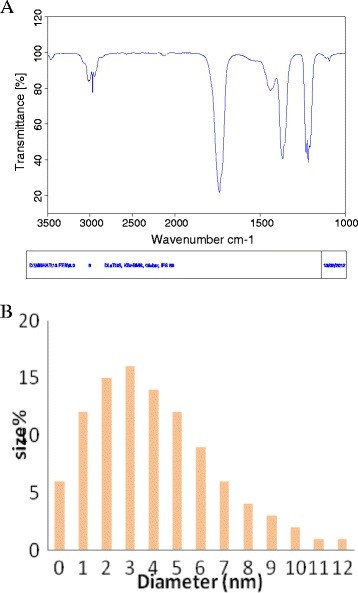
**(A) FT-IR spectra of the solution of the gold nanoparticles.(B)** Histogram indicating size distribution of nanoparticles formed after 22 hrs of incubation.

Transmission Electron Microscopy demonstrated a polymorphic distribution in size and shapes of gold nanoparticles formed by incubating gold chloride solution with whole cells of RS11. The two TEM images (Figure 2 C and 2 D**)** clearly show discrete gold nanoparticles of less than 20 nm size were formed and they were mostly spherical with occasional nano-triangles indicating that it was possible to synthesize gold particles of nano dimension with satisfactory level of mono-dispersity. FT-IR analysis of the reaction mixture has helped to understand the nature of biomolecules involved in the formation of gold nanoparticles. The FT-IR spectrogram **(**Figure 3**A)** of the reaction mixture has showed the presence of two bands at 3027 cm^-1^ and 2977 cm^-1^ which can be assigned to the stretching vibrational frequency of primary and secondary amines respectively. Three bands were observed in the FT-IR spectra at 1650, 1541 and 1441 cm^-1^. The first two bands i.e. bands at 1650 cm^-1^ and 1541 cm^-1^ are due to carbonyl stretch and the N-H bonds vibration respectively and are characteristic indicators of amide I and amide II linkages. The band at 1441 cm^-1^ may be assigned to methylene scissoring vibrations from the proteins in the solution. The bands observed in the spectra are similar to that of native proteins and also in agreement with the results obtained for gold colloid pepsin conjugates. Two bands at 1370 cm^-1^ and 1096 cm^-1^ have originated due to the C-N stretching vibration of aromatic and aliphatic amines. These bands altogether clearly indicate that the secondary structure of the proteins remains unaltered in presence of AuCl^4-^ ions and during formation of gold nanoparticles. The gold nanoparticles synthesized by RS11 were further characterized using Dynamic Light Scattering (DLS). Dynamic Light Scattering measurement of the AuNPs showed that the average particle size varied between 2–10 nm **(**Figure 3**B)**. It was observed that the average particle size initially was very high and decreases consistently to 24 h of incubation and remains unchanged thereafter. Overall analysis of the spectra indicated the presence of proteins in the sample which may have been used for reduction and/or capping and stabilization of the nanoparticles. Protein can bind to the nanoparticles either by interaction through its free amine group or by cysteine residues or through the amine groups present in the lysine residues. However, long term stabilization of the gold nanoparticles will obviously depend on the stability of the capping formed by proteins. An investigation on the time course of the AuNP formation as carried out by DLS revealed that AuNP formation started immediately after addition of the HAuCl_4_ solution to the cell suspension (3 mg/ml). Initially the particles obtained were of bigger size (> 20 nm), however, the particle size changed with the increase of incubation time resulting in formation of smaller particles. After 24 h of incubation particles with less than equal to 10 nm sizes were obtained. This indicated that as the time increases, due to the stimuli originated by the addition of HAuCl_4_ the presence of the capping material increased in the solution and capped the smaller nanoparticles efficiently. However, after prolonged incubation (more than 96 h of incubation), when the particle concentration reached a critical value, aggregate formation started. Therefore, an increase in the particle size was observed. On the basis of the observed time dependent change in the particle size it could be presumed that the quantity of capping materials become limiting factor and due to the availability of different quantity of capping material at different time there was a change in the particle size.

## Conclusion

Screening of several marine microbial strains revealed that the *Marinobactor pelagius* has the capability to synthesize AuNPs from HAuCl_4_ solution. The microorganism has been characterized taxonomically and a phylogenetic tree has been developed based on 16 S rRNA gene sequences showing the relationship of *Marinobacter* sp. RS11 with member of the genus *Marinobacter*. The AuNPs have been characterized UV-Visible spectroscopy, DLS and TEM. The result indicated that the microorganism can synthesize AuNPs less than 10 nm size (around 2 – 6 nm) with varied shape within a short period of time. The present work highlighted the possibility of using the marine bacterial strain of *Marinobacter pelagius* to achieve a fast rate of nanoparticles synthesis which may be of high interest for future process development of AuNPs which will enable harnessing marine water as a rich source of gold nanoparticles.

## Methods

The bacterial strains used in this study were isolated from the samples collected from marine habitats viz., solar-salterns, oceans etc. (Table 1). Samples were serial diluted and plated on marine agar plates and incubated at 30 °C for one week. Eleven unique morphotypes were picked and purified by repeated streaking on marine agar plates and were stored in 10% glycerol at −80 °C for long term maintenance. All of these bacteria were identified based on 16 S rRNA gene sequence analysis and screened for their capability to synthesize gold nanoparticles. The strain (RS 11) having capability of AuNP synthesis was selected for further characterization by polyphasic taxonomic approach. Cell morphology studies of the strain RS11 were done by phase contrast microscopy (olympus BX51, Tokyo, Japan) and also using Transmission electron microscope (JEOL 1200 EX II).

To screen AuNP synthesizing capabilities, all the isolates were grown on zobella marine broth (37 °C temperature and a shaker rpm of 200) and the cells were harvested by centrifugation (12,500 rpm, 4 °C for 15 min) once the cells reach the stationary phase. 10 mgs of washed cells (wet wt. basis) were incubated with 10 ml aqueous solution of HAuCl_4_ having a concentration of 250 mg/L. The pH of the final solution was adjusted between 5–6 using 0.1 M NaOH and 0.1 N HCL. The formation of gold nanoparticles was monitored by UV-Visible spectroscopy by recording the spectra between 300–650 nm and simultaneously monitoring the appearance of the characteristic peak of gold nano particles at 540 nm using a double beam spectrophotometer (Hitachi U-2900). Particle size measurement was done by carrying out experiments using DelsaNanoC (Beckman Coulter, USA). The instrument acquired scattering data at back angles (165°). About 1 ml of sample was used in disposable cells to acquire scattering data. The morphology of AuNPS was studied by TEM where carbon-coated copper grids were floated on a drop of the AuNPS aqueous suspension and blotted dry. Grids were then examined in a JEOL 2100 TEM operated at 200 KV. Representative fields were photographed. For 16 S rRNA gene sequencing, DNA was prepared using a bacterial DNA isolation kit (Genomic DNA kit (Qiagen).). The 16 S rRNA gene was amplified by PCR using universal bacterial primers 27f (5’-AGAGTTTGATCCTGGCTCAG-3’) and 1492r (5’-TACGGYTACCTTGTTACGACTT-3’). The PCR product was purified using QIA quick PCR purification kit (Qiagen, Germany) and it was sequenced using an ABI PRISM model 3700 automatic DNA sequencer and Big Dye Terminator cycle sequencing kit (Applied Biosystems, USA). The 16 S rRNA gene sequence was subjected to BLAST sequence similarity search [26] and Ez Taxon to identify the nearest taxa. All the nearest 16 S rRNA gene sequences were downloaded from the NCBI database (http://www.ncbi.nlm.nih.gov) and aligned using the CLUSTAL_X program [27] and the alignment was corrected manually using the **BioEdit** sequence alignment editor [28]. Phylogenetic tree was constructed using tree making algorithms, the Maximum likelihood (ML) and Neighbor joining (NJ) method [29] using MEGA5 [30].

## Competing interests

The authors declare that they have no competing interests.

## Authors' contributions

NS carried out experiments and helped in drafting of the manuscript. AKP isolated the strains and carried out taxonomic characterization of the selected microbes. MR helped in performing and TEM analysis and FNUA contributed in performing and analyzing DLS data. MS provided technical inputs in experiment designing and helped in drafting of the manuscript. RC conceived the study, designed the experimentation and drafted the manuscript. All authors read and approved the final manuscript.
